# Predictors of Survival Following Acute Nontraumatic Mesenteric Ischemia in North India: A Prospective Observational Study

**DOI:** 10.7759/cureus.107066

**Published:** 2026-04-14

**Authors:** Vivek K Bhagat, Ashok K Puranik, Satya Prakash Meena, Mayank Badkur, Indra S Choudhary, Mahendra Lodha, Ramkaran Chaudhary, Naveen Sharma

**Affiliations:** 1 General Surgery, All India Institute of Medical Sciences, Jodhpur, Jodhpur, IND; 2 General Surgery, All India Institute of Medical Sciences, Guwahati, Guwahati, IND

**Keywords:** crp, ldh, mesenteric ischemia, mortality, predictors of survival

## Abstract

Introduction: Acute mesenteric ischemia (AMI) is a life-threatening and rare cause of abdominal pain. No single laboratory test or clinical finding helps diagnose or predict the prognosis of AMI.

Aim: This study aimed to determine the morbidity and prognostic factors that affect the postoperative outcomes of patients undergoing surgery for bowel ischemia.

Methodology: This prospective observational study was conducted between July 2019 and December 2020 in North India. The study included 40 patients undergoing surgery for bowel ischemia. Patients under 18 years of age and those with bowel ischemia following trauma were excluded.

Results: A total of 40 patients were treated, out of whom 15 (37.5%) survived. Overall mortality was 62.5% (25/40). The mean age of the study population was 56.18 years. Compared with survivors, arterial pH was significantly lower (7.36 vs. 7.40, *P* = 0.031), C-reactive protein (CRP) levels were higher (194.30 mg/L vs. 128.70 mg/L, *P* = 0.017), and lactate dehydrogenase (LDH) levels were also elevated in deceased patients (471.22 U/L vs. 335.80 U/L, *P* = 0.016). Distal to the duodenojejunal (DJ) junction, the remaining small bowel length was significantly shorter in deceased patients than in survivors (45 cm vs. 142 cm, *P* < 0.001). All six patients (15%) who had large bowel involvement died. Postoperative complications included relaparotomy in 15 patients (37.5%), anastomotic leak in 6 patients (15%), and requirement of ventilatory support in 16 patients (40%).

Conclusions: AMI continues to carry a high risk of mortality, particularly in patients presenting late with extensive bowel involvement and systemic complications. The use of antiplatelet drugs is an effective way to prevent severe complications. Patient age, pH, LDH, CRP, serum lactate, remaining length of small bowel from DJ, and large bowel involvement are significant predictors of mortality.

## Introduction

Abdominal pain caused by acute mesenteric ischemia (AMI) is rare and life-threatening. The diagnosis of mesenteric ischemia remains challenging, with reported incidence rates between 0.09% and 0.2% of all surgical admissions [1]. Diagnosis requires high clinical suspicion as no single laboratory test is definitive. The mortality rate associated with AMI remains high, despite medical advancements [2-3]. The late form of AMI, with irreversible transmural necrosis, is not reversible at all [4]. There are several causes of AMI, including superior mesenteric artery (SMA) thrombosis (15%-25%), embolism (50%), and venous thrombosis (5%-15%). These are often associated with atherosclerosis or emboli. In elderly patients, venous thrombosis is the most common cause of AMI with abdominal pain [4-5]. The study aimed to determine the prognostic factors and morbidity patterns of patients undergoing bowel ischemia surgery.

## Materials and methods

Study design and setting

A prospective observational study was conducted at the All India Institute of Medical Sciences (AIIMS), Jodhpur, North India, between July 2019 and December 2020.

Study population

Patients undergoing surgery for bowel ischemia caused by vascular thrombus or embolism were included. The study excluded all patients younger than 18 years of age and who had a history of trauma.

Sample size calculation

The sample size was calculated assuming a 50% mortality rate, 80% power, and a 5% alpha level, yielding approximately 40 patients using OpenEpi (Atlanta, GA).

Objectives

This study aimed to determine morbidity patterns among patients undergoing surgery for AMI. Secondary objectives were to assess the prevalence of bowel ischemia, identify risk factors, describe clinical presentation patterns, evaluate outcomes, and assess management options.

Procedure

Patients presenting with acute abdominal pain and suspected bowel ischemia, according to clinical or radiological assessment, underwent laparotomy. These patients received adequate hydration, antibiotics, analgesia, and were taken for exploratory laparotomy within six hours of presentation. Surgical procedures were performed based on operative findings and the patient’s condition. Postoperatively, all patients received low-molecular-weight heparin (LMWH), low-dose aspirin, total parenteral nutrition, and warfarin. Vascular interventions were not performed during hospitalization due to late presentation.

Ethical approval and informed consent

Informed consent for the treatment and publication was obtained from all participants in this study. Ethical approval was obtained from the Institutional Ethics Committee (AIIMS/IEC/2019/1833). All authors declared that no financial support was received from any organization for the submitted work.

Data collection

Demographic information, drug history, comorbidities, and other relevant parameters were collected. Preoperative baseline and diagnostic investigations, postoperative serum electrolytes, nutritional requirements, surgical interventions, hospital stay, and timing of stoma closure were documented.

Statistical analysis

The data were analyzed using SPSS Statistics software, version 25 (IBM Corp., Armonk, NY). Descriptive statistics, chi-square tests, Mann-Whitney U tests, and t-tests were used for analysis. Parametric tests (independent/unpaired t-test) were applied to continuous variables with a normal distribution, while non-parametric tests (Mann-Whitney U test) were used for skewed data or variables not following normality. Categorical variables were analyzed using the chi-square test or Fisher’s exact test. Statistical significance was determined by a *P*-value of 0.05.

## Results

Demographic and baseline characteristics

**Figure 1:**
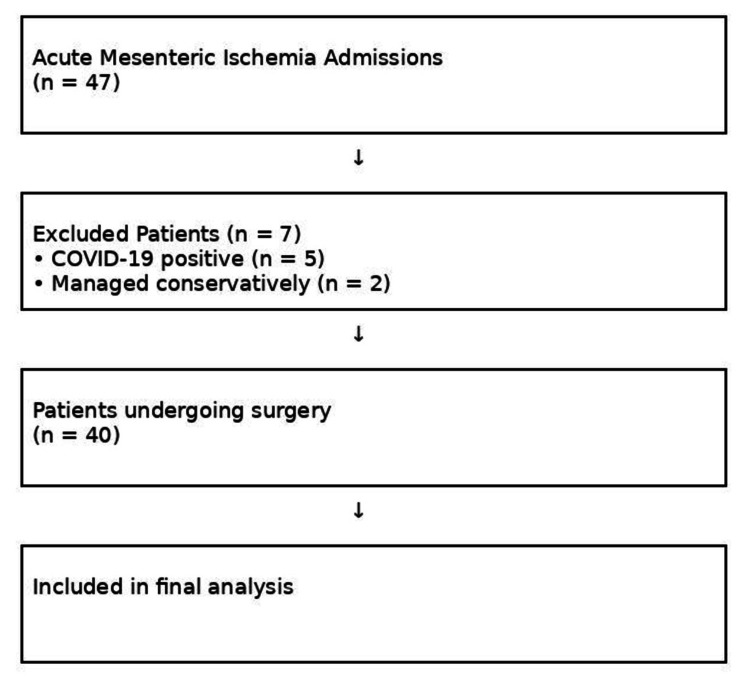
Flow diagram. Image credit: All authors.

Out of 47 patients, 40 (85.1%) underwent surgery for AMI (Figure 1). 

Compared to survivors, non-survivors had a higher average age. In both groups, males were predominant, with no statistically significant difference between genders. Diabetes mellitus and hypertension were more common among non-survivors, though these differences were not statistically significant. Most non-survivors showed signs of shock and peritonitis upon admission. Non-survivors also had significantly worse metabolic status, as indicated by lower arterial pH and higher inflammatory markers, according to laboratory data (Table 1).

**Table 1:** Demographic data analysis of the mesenteric ischemia patients (n=40) DM, diabetes mellitus; HTN, hypertension; COPD, chronic obstructive pulmonary disease; PVD, peripheral vascular disease; CHF, congestive heart failure; CAD, coronary artery disease

Data	Number of patients (n = 40)	Percentage (%)
Demographic characteristics		
Male	27	67.5
Female	13	32.5
DM	10	25
HTN	18	45
COPD	4	10
PVD	5	12.5
CHF	1	2.5
CAD	7	17.5
Anticoagulant drug history		
Aspirin	4	10
Clopidogrel	4	10
Heparin	2	5
Warfarin	2	5
Addiction		
Alcohol	5	12.5
Tobacco smoking	14	35
Tobacco chewing	3	7.5
Opium	4	10

In this study, 26 (65%) patients had a history of addiction to opium, smoking, or tobacco chewing. Overall, 12 (30%) patients were receiving anticoagulants for various indications. Among the participants, 34 (85%) had superior mesenteric artery (SMA) thrombosis and 6 (15%) had superior mesenteric vein (SMV) thrombosis as the cause of AMI.

Biochemical and laboratory comparisons

Several biochemical markers were significantly elevated in non-survivors, indicating their potential role in prognostication. Elevated BUN, creatinine, triglycerides, D-dimer, amylase, and lipase were associated with increased mortality. These findings suggest that deranged renal parameters and pancreatic enzymes could reflect the extent of ischemic injury and systemic inflammatory response, warranting their routine assessment in patients with bowel ischemia (Table 2). 

**Table 2:** Hematological and biochemical parameters of patients with mesenteric ischemia (n = 40). Test statistic: Unpaired t-test value calculated as *t* = (*M*₁ - *M*₂)/√[(SD₁²/*n*₁)+(SD₂²/*n*₂)]. *P < 0.05 (statistically significant). Hb, hemoglobin; WBC, white blood cell count; BUN, blood urea nitrogen; INR, international normalized ratio; SGPT, serum glutamic pyruvic transaminase; SGOT, serum glutamic oxaloacetic transaminase; HDL, high-density lipoprotein; LDL, low-density lipoprotein

Parameter (Mean ± SD)	All patients (40) (Mean ± SD)	Alive (15) (Mean ± SD)	Dead (25) (Mean ± SD)	Reference range	Test statistics	*P*-value
Hb (g/dL)	12.24 ± 2.77	11.94 ± 2.85	12.42 ± 2.71	12-16	*t* = 0.82	0.212
WBC (cells x10^3^)	15.63 ± 7.55	15.38 ± 7.82	15.78 ± 7.42	4-11	*t* = 0.31	0.388
BUN (mg/dL)	60.60 ± 39.70	48.47 ± 32.15	67.88 ± 42.36	7-20	*t* = 2.35	0.021*
Creatinine (mg/dL)	1.50 ± 0.92	1.22 ± 0.78	1.66 ± 0.98	0.6-1.2	*t* = 2.15	0.034*
INR	1.48 ± 0.41	1.48 ± 0.42	1.48 ± 0.40	0.8-1.2	*t* = 0.00	0.997
SGPT (U/L)	35.97 ± 25	40.16 ± 24.50	33.46 ± 25.80	10-40	*t* = 1.52	0.141
SGOT (U/L)	48.08 ± 46.49	40.32 ± 38.24	52.74 ± 50.92	10-40	*t* = 1.75	0.085
Bilirubin (mg/dL)	1.39 ± 1.70	1.28 ± 1.62	1.69 ± 1.75	0.3-1.2	*t* = 1.22	0.072
Albumin (g/dL)	3.04 ± 0.71	3.15 ± 0.72	2.98 ± 0.70	3.5-5.0	*t* = 0.89	0.119
Cholesterol (mg/dL)	101.00 ± 40.94	100.73 ± 41.28	101.16 ± 40.82	<200	*t* = 0.07	0.812
Triglyceride (mg/dL)	178.30 ± 112.97	138.4 ± 98.54	202.24 ± 118.62	<150	*t* = 2.44	0.018*
HDL (mg/dL)	25.35 ± 14.09	25.6 ± 14.32	25.2 ± 13.95	>40	*t* = 0.12	0.640
LDL (mg/dL)	57.78 ± 27.14	61.47 ± 27.85	55.56 ± 26.68	<100	*t* = 1.21	0.238
D-dimer (µg/mL)	6.07 ± 4.66	5.13 ± 4.02	6.63 ± 5.01	<0.5	*t* = 2.06	0.041*
Amylase (U/L)	129.13 ± 117.26	93.8 ± 95.23	150.32 ± 128.45	30-110	*t* = 2.25	0.028*
Lipase (U/L)	84.60 ± 149.07	68.07 ± 132.45	94.52 ± 158.23	13-60	*t* = 2.10	0.039*

Intraoperative findings

All patients diagnosed with AMI underwent exploratory laparotomy within six hours of their arrival at the surgical emergency department. In two patients, only congested small bowel was visible during laparotomy; it was therefore irrigated with warm saline, and the abdomen was left open for reinspection after 36-48 hours. One patient died immediately in the postoperative period due to septic shock, and another patient had their abdomen closed and was discharged, but died within a week of leaving the hospital. The cause of death was unknown.

The majority of patients (26/40, 65%) underwent proximal stoma formation-either duodenostomy, jejunostomy, or ileostomy-along with a distal mucous fistula following resection of the affected small bowel segment. Combined resection of both the small and large bowel due to gangrenous involvement was performed in six patients (15%). In two patients (5%), observation alone was undertaken as bowel viability improved with restoration of normal color. Postoperative complications requiring re-exploration occurred in seven patients (17.5%), primarily due to stoma gangrene or progression of bowel gangrene. Primary side-to-side anastomosis following resection of gangrenous small bowel was performed in six patients (15%) (Table 3).

**Table 3:** Intraoperative findings and procedures performed in patients with mesenteric ischemia (n = 40).

Procedure	Intraoperative findings	Numbers of patients	Percentage
Resection of the jejunoileal segment with proximal stoma formation and distal mucous fistula	Gangrenous small bowel	26	65
Resection of the jejunoileal segment with primary anastomosis	Gangrenous small bowel	6	15
Resection of both the small and large bowel	Gangrenous involvement of the small and large bowel up to two-thirds of the transverse colon	6	15
Warm saline wash for congested small bowel	Only congested small bowel	2	5

Postoperative outcomes and prognostic factors

In the postoperative period, six patients (15%) developed anastomotic leakage, two of whom occurred after stoma closure. A total of 15 patients (37.5%) underwent relaparotomy. Sixteen patients (40%) required ventilatory support and ICU care due to postoperative complications. The mean length of hospital stay was 28 days. Significant associations were found between mortality and postoperative complications, including sepsis, septic shock, acute kidney injury, bloodstream infection, chest infection, thrombocytopenia, relaparotomy, and dyselectrolytemia. These outcomes suggest that prompt identification and management of these complications are critical in reducing postoperative mortality following bowel ischemia (Table 4).

**Table 4:** Postoperative outcomes of mesenteric ischemia, including the comorbidities of patients (n = 40). Test statistic: χ² (chi-square) derived from a 2 × 2 contingency table. *P < 0.05 (statistically significant).

Postoperative outcome	Number of patients (n = 40)	Alive (n = 15)	Dead (n = 25)	Test statistic	*P*-value
Sepsis	22 (55%)	2	20	*χ*² = 14.25	<0.001*
Septic shock	14 (35%)	0	14	*χ*² = 10.58	<0.001*
Acute kidney injury (AKI)	19 (47.5%)	10	9	*χ*² = 4.37	0.037*
Re-laparotomy	15 (37.5%)	6	9	*χ*² = 5.06	0.024*
Surgical site infection (SSI)	14 (35%)	6	8	*χ*² = 0.03	0.116
Hepatic dysfunction	13 (32.5%)	5	8	*χ*² = 0.15	0.084
Dyselectrolytemia	13 (32.5%)	4	9	*χ*² = 3.92	0.048*
Chest infection	11 (27.5%)	2	9	*χ*² = 5.50	0.019*
Blood infection	10 (25%)	1	9	*χ*² = 6.38	0.012*
Anastomotic leak	6 (15%)	3	3	*χ*² = 0.05	0.998
Thrombocytopenia	6 (15%)	0	6	*χ*² = 7.11	0.007*
Urinary tract infection (UTI)	5 (12.5%)	2	3	*χ*² = 0.00	0.332
Anasarca	3 (7.5%)	1	2	*χ*² = 0.00	0.412

Patient age, pH value, CRP, LDH, bowel resection length, remaining bowel from the duodenojejunal (DJ) junction, and antiplatelet drugs were significantly associated with mortality. People who survived were younger than those who died, and those with decreased pH had an increased risk of death. Patients who underwent resection of a mean length of 195 cm of small bowel and had a remaining small bowel length of ≤45 cm from the DJ junction did not survive (Table 5). Among the study participants, 15 (37.5%) survived, while 25 (62.5%) did not.

**Table 5:** Significant prognostic factors of mesenteric ischemia in the study (n = 40). Independent t-test (parametric, assuming normality), Mann-Whitney U test (non-parametric, for bowel lengths), and χ² test or Fisher’s exact test (for categorical variables). Test statistics: t-value, U-value, or χ² value. CRP, C-reactive protein; LDH, lactate dehydrogenase; DJ, duodenojejunal junction *P < 0.05 (statistically significant).

Significant parameter	All (*n* = 40) (Mean ± SD)	Alive (*n* = 15) (Mean ± SD)	Dead (*n* = 25) (Mean ± SD)	Test statistic	*P*-value
Age (years)	56.18 ± 10.5	49.20 ± 9.8	60.36 ± 9.2	*t* = -2.77	0.007*
pH	7.37 ± 0.86	7.40 ± 0.35	7.37 ± 1.12	*t* = -2.20	0.031*
CRP (mg/dL)	169.70 ± 92.90	128.70 ± 78.45	194.30 ± 98.23	*t* = -2.45	0.017*
Lactate (mmol/L)	2.75 ± 1.72	1.78 ± 1.12	3.33 ± 1.92	*t* = -2.97	0.004*
LDH (U/L)	420.75 ± 175.96	335.80 ± 142.37	471.22 ± 187.23	*t* = -2.46	0.016*
Small bowel resected (cm)	163.46 ± 85.2	113 ± 75.4	195 ± 82.1	*t* = -2.25	0.027*
Residual length DJ (cm)	82.31 ± 65.3	142 ± 52.8	45 ± 38.7	*U* = 85	<0.001*
Sepsis	22 (55%)	2	20	*χ*² = 14.25	<0.001*
Septic shock	14 (35%)	0	14	*χ*² = 10.58	<0.001*
Aspirin use	4 (10%)	4	0	*χ*² = 7.41	0.006*
Clopidogrel use	4 (10%)	4	0	*χ*² = 7.41	0.006*

## Discussion

Despite advancements in medical technology, the mortality rate following AMI remains very high. Improving patient outcomes requires a better understanding of the morbidity and mortality patterns associated with bowel ischemia.

In this study, 34 (85%) of mesenteric ischemia cases were due to SMA thrombosis. This is higher than that reported in the literature. Kuhn’s study showed that small intestinal hypoperfusion can be induced by various etiologies, including mesenteric arterial embolism (50%), mesenteric arterial thrombosis (15%-25%), and mesenteric venous thrombosis (5%-15%) [6]. In a retrospective study by Kase et al., among 577 patients with AMI, 60% had superior mesenteric artery involvement (54% thrombosis and 12% embolism) as the cause, 7% had inferior mesenteric artery thrombosis, 7% had non-occlusive mesenteric ischemia, 4% had mesenteric venous thrombosis, and the remainder had an unclear etiology [7]. SMA thrombosis occurs more frequently at its origin, leading to greater ischemia, bowel infarction, necrosis, and extensive bowel involvement from the duodenum to the transverse colon. In this study, no cases of mesenteric arterial embolism were identified as a cause of AMI. This contrasts with the literature, which frequently implicates arterial embolism as a major cause of AMI. Mesenteric arterial embolism is reported as the most common cause of AMI, accounting for 40% to 50% of cases in the study by Liao et al. [8].

Among the 40 patients included in this study, 25 died during the study period, yielding a mortality rate of 62.5%. Tamme et al.'s study reported long-term mortality due to mesenteric ischemia and short-term mortality following intervention to be 68% and 34%, respectively [9]. Adaba et al.'s systematic review reported an in-hospital mortality of 63% among 4,527 patients, with no difference in mortality between occlusive and non-occlusive mesenteric arterial causes [10]. The mortality rate of 62.5% observed in the present study is comparable to rates reported in other studies, particularly in the Indian context, where delayed presentation to the hospital contributes to higher overall mortality associated with AMI.

Despite its rarity, AMI occurs in 0.09% to 0.2% of surgical patients [9-11]. Stoncy and Cunningham reported a higher incidence of 1 per 1,000 hospital admissions [12]. A recent study by Crawford et al., titled “A Statewide Analysis of the Incidence and Outcomes of AMI in Maryland from 2009 to 2013,” similarly reported approximately one admission per 1,000 hospital admissions annually [13]. During the study period from July 2019 to December 2020, there were 1,227 surgical emergency admissions. Based on existing literature, approximately one case of acute mesenteric ischemia would be expected.

During the same period, a total of 47 AMI cases were admitted to the institute. The institute caters to the surgical needs of a large population in western Rajasthan. It is difficult to estimate the exact population covered or the true disease prevalence in this study. However, the observed incidence was approximately 30 times higher than expected.

In the postoperative period, complications related to the stoma site included stoma gangrene, skin excoriation, high-output stoma, and issues with enteroclysis. Patient age was significantly associated with mortality, with survivors being younger than those who died. The mean age of patients who survived after surgery was 49.20 years. In a study by Park et al., patients under 60 years of age had a mortality rate of 12%, while those over 60 years had a mortality rate of 54% in AMI [14]. In a retrospective study by Acosta-Merida et al., the mean age of patients who survived was 71 years, while it was 75 years among those who died [15].

The patient profile in the present study was approximately 10 years younger at the time of AMI diagnosis. This was also reported in a study by Kundan et al., where most patients were between 50 and 59 years of age, with increasing age associated with higher mortality [16].

Several studies have shown that pH levels are significantly associated with mortality, with lower pH levels indicating a higher risk [15,17]. The findings of the present study were consistent with those of other studies.

A study by Mishra et al. concluded that elevated lactate levels were associated with mesenteric ischemia [18]. A lactate level >2 mmol/L was associated with irreversible intestinal ischemia. Patients with normal mean lactate levels (<2 mmol/L) had a better prognosis.

Luwaga et al.’s study showed that participants with irreversible bowel ischemia had increased serum lactate levels than those in the reversible and non-ischemic groups, indicating that serum lactate has moderate sensitivity for bowel ischemia [19]. Kacer et al. found that a CRP/albumin (CRP/Alb) ratio >1.32 was significantly associated with AMI [20]. In a study by Salem et al., increased CRP levels were observed in patients with AMI [21].

A study by Nagaraj et al. showed that patients who survived had a mean of 91 cm of small bowel resected, while those who died had a mean length of 152 cm. The mean length of small bowel resected in patients who survived in this study was 113 cm, consistent with previous findings [22]. In a study by Kundan et al., a 90.90% mortality rate was reported in patients with a functional bowel length of 1 foot or less, which aligns with the findings of the present study.

A study by Fuglseth et al. found that antiplatelet drugs are preventive in the non-operative treatment of chronic mesenteric ischemia [23]. This contrasts with the present study’s findings, which showed that all patients taking antiplatelet therapy survived. This disparity may be due to other confounding factors, such as patient age and the length of viable small bowel distal to the DJ junction, which may influence mortality in the postoperative period.

For patients who develop sepsis with or without shock in the postoperative period, there is a statistically significant increase in the risk of mortality. Some studies have shown that shock is significantly associated with morbidity and mortality [17,24]. The findings of this study are consistent with previous research.

Limitations

This single-center study at AIIMS Jodhpur (*n* = 40) limits generalizability. The lack of preoperative vascular imaging in all patients prevented precise differentiation of etiology. Short follow-up limited assessment of long-term outcomes. The small sample size precluded multivariate analysis. The lack of bowel transplant facilities in India restricted short bowel syndrome management options for extensive resection cases (<45 cm residual bowel), potentially worsening mortality (62.5%). No standardized ischemia timing or serial biomarkers have been shown to improve prognostic accuracy. Resource constraints and the typical late presentations in North India further limit broader applicability.

## Conclusions

Mesenteric ischemia has a high mortality rate, with superior mesenteric artery thrombosis being the most common cause. Prognostic factors include advanced patient age, acidosis, elevated CRP and lactate levels, elevated LDH, extensive small bowel resection, and minimal residual small bowel length distal to the duodenojejunal junction (<45 cm). Postoperative sepsis and septic shock strongly predict poor outcomes, while antiplatelet medication use correlates with improved survival. Extensive bowel involvement, particularly involving the large bowel, severely limits survival prospects. A proactive approach to recognition, resuscitation, and complication management remains crucial.
